# The Intra-Vascular Injection of Blood and Other Fluids

**Published:** 1885-05-20

**Authors:** Joseph C. Hutchison

**Affiliations:** Brooklyn, N. Y.


### THE INTRA-VASCULAR INJECTION OF BLOOD AND OTHER FLUIDS.

BY JOSEPH C. HUTCHISON, M.D., LL.D., of Brooklyn, N. Y.

Notwithstanding the operation of transfusion of blood has been practiced for more than five hundred years, and many brilliant illustrations of its value in saving human life have been noted, it must be reluctantly admitted that it has not fulfilled its early promise, and is now resorted to much less frequently than formerly.

The writer has, for a long time, believed that transfusion would be more frequently practiced, and take a higher rank as a therapeutic expedient, if the means for performing the operation were simplified, its dangers diminished, and a proper and easily obtainable fluid could be found for the purpose. The object of this paper is to consider these requirements.

### DESCRIPTION OF THE APPARATUS.

The apparatus which I have devised consists of a cylindrical glass receiver, graduated into inches, with a capacity of twelve ounces. A metal cap, containing a perforated female screw, is attached to its lower end, having a perforated, nipple shaped termination, to which a rubber tube four feet long is connected, in order to convey the liquid from the receiver to the canula which enters the blood-vessel.

The receiver is surrounded by a double jacket of India-rubber, the walls of which are separated by a half-inch space for holding hot water, and is arranged to admit a thermometer.

The apparatus is warmed by filling it with hot water, which is allowed to run off before it is filled with the fluid which is to be injected. The receiver is then closed with the rubber stopper carrying the thermometer, and the apparatus is suspended, or held by an assistant, three or four feet above the vein which is to be opened.

The rapidity with which the fluid is introduced can be regulated by the stop-cock in the canula. After the canula is introduced it is fixed in position by the finger laid over the opening in the vein; no ligature is necessary to retain it in position.

A vein at the bend of the arm is usually selected for the operation, a saphena near the outer ankle may be selected, or the fluid may be introduced into the radial artery, as recommended by Hunter, and recently practiced by Halsted. The central end of the artery should be selected.

A number of appliances have been devised for the purpose of simplifying the operation of transfusion, and for preventing the entrance of air into the blood-vessels; these results can be best obtained by a simple apparatus, constructed on the principle of the one I have exhibited. If the operation is managed with ordinary care, it is impossible for air to enter the vein. The special advantage which this apparatus has is the hot-water jacket. It is important that the injection should not enter the blood at less than 98.5° F., and there is no objection to temperature of 105° F. The temperature can be accurately regulated by the thermometer.

### TRANSFUSION OF BLOOD.

It was believed, until recently, that the reviving power of blood resided in the corpuscles, that transfusion, when performed with the serum alone, or with any other fluid, would prove fruitless. Recently, however, Ott and other observers have demonstrated that the blood corpuscles is not the important element; that the indication is to restore activity to the circulation by restoring the bulk of the vital fluid so as to enable the organs, especially the heart and brain, to perform their functions duly; that, for these and other reasons, blood is not so good as saline solutions for transfusion in acute anæmia.

### SALINE INJECTIONS INTO THE VEINS.

The intra-venous injection of saline solutions was extensively practiced during the cholera epidemic of 1832—'33. More recently this matter has received considerable attention, especially by the Germans; and they have proved, by numerous experiments on animals bled to syncope, that life can be restored by injecting saline solutions into their veins, and that they are even to be preferred to blood for that purpose.

Dr. Hutchison then refers, at some length, to experiments performed by Ott, Schwarz, Jennings, Mikulicz, and a paper by Bull.

There are many nice points connected with the operation which can only be settled by experiment, such as the best method of introducing the liquid, the proper composition, temperature, specific gravity, quantity, and the rapidity with which it should be introduced. In two cases I injected a fluid composed of common salt, ʒ iij, and alcohol, ʒ j, in one pint of water, of which two pints were introduced into the median basilic vein, at a temperature varying from 100° to 110° F., and repeated the operation when the algid symptoms reappeared. The following solution, recommended by Dr. Gull in the "Reports on Epidemic Cholera to the Royal College of Physicians," London, 1854, was used in three cases:

Chloride of sodium, 69 parts by weight; chloride of potassium, 6 parts by weight; phosphate of sodium, 3 parts by weight; carbonate of sodium, 2·13 parts by weight.

"By dissolving one hundred and forty grains of this salt in forty ounces of distilled water, and filtering, we obtain a fluid having a decidedly saline taste, a faintly acid reaction, and nearly approximative in its composition to the fluid effused, minus the organic substances. These are small in amount, and their loss has, apparently, no important influence on the constitution of the blood."

In Dr. Gull's formula the sulphate of sodium is strangely omitted. A more accurate formula (the one which I recommend), deduced from Schmidt's analysis, is the following:

Chloride of sodium, 60 grains; chloride of potassium, 6 grains; phosphate of sodium, 3 grains; sulphate of sodium, 1·5 grains; carbonate of sodium, 20 grains.

Dissolve one powder in 24 ounces of water at 10.5° F. The fluid should enter the vein at a rate not exceeding one ounce per minute, and this can be accurately regulated by means of the stopcock in the canula. The quantity of fluid to be injected, if a saline solution is used, is from 12 to 24 ounces. The injection may be stopped when the pulse becomes stronger and the temperature of the peripheral parts of the body more normal.

The matter presented in the preceding pages seems to the writer to justify the following conclusions:

1. It appears to be proved, by experiments upon animals and by clinical facts, that corpuscles of transfused blood are short-lived and rapidly excreted; that the reviving power of blood does not reside in the red corpuscles, and hence the danger of excessive loss of blood is not due to the diminution of its corpuscles and other solid constituents.

2. The important element in transfusion is the restoration of the vital fluid to the vascular system, increasing vascular tension, and causing energetic contractions of the heart.

3. The intra-venous injection of salt solutions in appropriate cases is a more simple and safer operation than transfusion of blood. It can be done without the aid of a skilled assistant, and the materials for injection are easily obtained.

4. If further experience should confirm the favorable results from intra-venous injections that have been recently reported at home and abroad, the operation deserves to be held in the highest esteem, and is destined to occupy an important position among therapeutic agents.—*New York Med. Jr.*; *Epitome.*

---

**Ipecac in Dyspepsia.**—Dr. Fothergill, in *Medical Times*, writes: "A little bismuth with soda and *nux-vomica* is a good combination, so far as medicines are concerned, to commence with. But ipecacuanha is the drug *par excellence* when it can be borne. That this is an hepatic stimulant of no mean power is now generally admitted. It is notorious that in full doses ipecacuanha is an emetic. In smaller doses it increases the activity of the gastric secretion, while stimulating the muscular fibres of the stomach. Consequently it is a drug of great potency in cases of deficient power in the digestive organs; and, as a pill, two-thirds of a grain of ipecacuanha, with a dose of strychnia, a little black pepper in extract of gentian, or aloes and myrrh pill, or compound colocynth pill, according to the state of the bowels, is in constant use with me in cases of indigestion. By it before meals, and pepsin pills after, the number being regulated by the patient's necessities, and a suitable regimen, ordinary cases of indigestion can be made to move on satisfactorily. But there is a lower stratum than this, viz: a class of cases where only the most easily digested foods can be taken at all—where the stomach cannot be bullied, and where compromise alone is practicable. In these very troublesome cases 'baby-foods' are our sheet-anchor to start with. And even when something approaching an ordinary meal can be taken in small quantities, a glass of milk, with some good malt extract in it—about two hours after the meal, when the contents of the stomach are passing the pyloric ring—is a good addition to it. The increasing failure of digestive power of the present day calls into action the skill of the chemist; and fortunately for dyspeptics, the chemist posseses the skill."

